# Cases of Aveolar Abscess

**Published:** 1856-01

**Authors:** 


### CASES OF ALVEOLAR ABSCESS.

[In a former number of the *Register* we gave the treatment by Dr. Ballard for the cure of Alveolar Abscess. We give the report now of several cases which he has given in detail. We are satisfied that the treatment is based on correct views, and will very often succeed.—Ed.]

In the January number of the Recorder, we promised to furnish our readers with the history of some cases of Alveolar Abscess, illustrative of the plan of treatment advocated by us some months before. The course of treatment alluded to was founded upon the belief, that by far the greatest proportion of these abscesses were the result of decomposed or decomposing substances contained in the pulp cavity or nerve canal of the tooth. That this decomposition gave rise to matters which must somewhere find vent, and that when no exit could be had from the crown of the tooth, they forced their way through the foramen at the extremity of the fang, causing more or less inflammation of the periosteum of the tooth at that point; that at this stage of the disease, an effort was made by nature to repel and confine the discharging substances to the cavity of the tooth, and that where the amount of decomposition was trifling, this effort was often successful; the periosteum becoming thickened and indurated, and thus closing effectually the foramen of the root, but that when this attempt at a *natural cure* failed, the membrane surrounding the fang became the sac of the abscess—that this eventually opened, and a sinus being formed through the alveolar process and gum, the discharge was thus made, continuing for an indefinite period, increasing and diminishing from time to time, as exciting or soothing influences were brought to bear upon the affected organ and its surrounding tissues.

The course of treatment advocated was, first—removal of all decomposed substances contained in the pulp cavity of the tooth; second—to arrest effectually the process of decomposition both in the nerve cavity and beyond it. The first to be accomplished by the use of such instruments as could be readily passed into the canal to its extremity, and by repeated and thorough washing with water (by means of a syringe.) The second object to be attained by the use of creosote. This substance is well known as an anti-septic; it will prevent the decomposition of animal substances; it will do more, it will arrest decomposition after it has commenced, and still more, for it will often restore animal matter which has become slightly decomposed or "tainted." Meat held for a few minutes in a weak solution of creosote and water can be hung up in the air till dried and is in effect cured or smoked —at least so far as its preservation is concerned.

It is a matter of daily notice that teeth with dead nerves in them turn dark; it is simply because their decomposed contents have entered the tubules forming the dentine; to remove all of this discolored bone would greatly weaken the tooth. Fortunately, it is seldom necessary. The removal of comparatively a small portion will, aided by the yellow tinge caused by the subsequent filling with gold, generally prove sufficient to restore the natural appearance or so near it as to escape notice. Still there yet remains a large amount of dentine, the tubes of which contain decomposed matter; to leave this so and fill the tooth and fang would only insure a darker shade to the tooth. In the majority of cases a very decided change of color would be noticed after a few weeks had elapsed.—We know of no way of obviating this difficulty except by treating the contents of the dentinal tubes with creosote.

The tubes cannot be emptied—we cannot purge them of their disgusting and baneful contents, but we can leave the decomposed substances in their resting-place in a pure and inert condition, so that they will neither injure nor discolor the tooth. This end we accomplish by means of creosote and with more certainty than with any other agent with which we are acquainted. So much for preface. We are now prepared to make some practical illustrations.

---

Case I.—March 16th, 1854.—Miss M——, aged 16. Constitution feeble, with scrofulous taint. Teeth very large—crowded—incisiors over-lapping. The whole denture composed of teeth of very soft character, and at this time very badly decayed. The rlght superior lateral incisor has the nerve exposed. The adjoining central contains the remains of a dead nerve—large abscess at the extremity of the fang, and crown of tooth very much discolored. The left lateral incisor very deeply decayed upon both approximal surfaces,

Made an application of arsenic and creosote to the exposed nerve of lateral incisor. Cleaned nerve cavity of right central, and filled the fang with floss silk, moistened with creosote.

17th.—Removed nerve from the lateral, arrested the hemorrhage, and filled the fang with gold foil. Renewed the application of creosote in nerve cavity of the central.

18th.—Filled the crown cavity of the lateral, and again renewed the application to the central. Abscess does not seem to have changed, though the exhalations from the nerve cavity are of a much improved character, giving but a slight ammoniacal taint to the silk floss with which it had been filled.

Between this time and the 22d, the application was renewed several times, and the nerve of the left central incisor having been exposed too much to be filled successfully by capping, (we attempted it but failed,) we destroyed the nerve and filled the root with gold. At this date, finding no taint of anything but creosote upon the floss with which the nerve cavity of the right central was filled, it was removed, the canal thoroughly washed out and dried, and then filled with gold. The abscess discharging less but still remaining.

24th.—Filled crown cavity of right central. Abscess still open.

25th.—Filled a crown cavity in the left central, but not the one opening into the pulp chamber. We omitted to state that great trouble was caused by the hemorrhage from the nerve vessels of this tooth; it seemed almost impossible to arrest it. We filled it with floss saturated with camphor, and allowed it to remain twenty-four hours; upon removing the filling the hemorrhage re-commenced, but by injecting cold water it was at length controlled, as we supposed, and the root filled with gold. We did not see the patient for some weeks after the 25th, but learned from members of the family that she had been quite ill—confined to her bed, and that her face had been very much swollen.

April 18th.—Patient again made her appearance. Stated that one of her teeth was quite loose—it had caused her much suffering, and her face had swollen very much—that these unpleasant symptoms commenced the evening of the 25th, but had eventually disappeared after a copious discharge of matter, leaving the tooth quite sore and loose. Upon examination found that an abscess had opened immediately over the left central incisor, and that this was the tooth that had caused the trouble. The abscess connected with the right central had disappeared entirely; its former position being marked by a small spot somewhat redder than the tissue around it.—This tooth was firm and entirely free from pain or soreness, there being no unpleasant sensations connected with it.

From this state of things we judged that the hemorrhage from the nerve vessels of the left incisor had re-commenced after the fang was filled, and probably was caused by the jar and pressure of filling the crown cavity, as there was no soreness or apparent trouble until after this was filled. We cannot but consider this case as a most convincing one, so far as the treatment advocated is concerned. It not only was entirely healed by the applications, but all this, i. e. the actual healing and closing up of the abscess, was accomplished while there was a great amount of local irritation and disease connected with the adjoining tooth, and sufficient as the result proved to establish an abscess over that tooth. There could be no connection between the two teeth that could have caused the discharge from the right central to come out over the left one. Had there been any chance of a connection, the new opening would not have occurred, nor the old one have closed. It may be of interest to know that the operation performed upon the right lateral incisor was entirely successful.

June 2d.—Finished the operation upon the left central, by filling the cavity leading to the canal of the tooth. There is a slight opening over the root of this tooth—no perceptible discharge—no soreness—tooth quite firm.

Sept., 1855.—No signs of abscess over either of the teeth. Patient states that she has had trouble once since we saw her last—does not know which tooth; there was considerable soreness, and one of the teeth felt loose, but no discharge or swelling.

Case II.—Feb., 1852.—This patient was a young man aged 21, in good health, florid complexion, active habits.—Called while in great suffering from a superior right central incisor. Had been troubled with it for two or three days, but not so severely or constantly as during the night previous. The tooth was firm in the socket, but pressure upon it caused intense pain that seemed to affect the whole face. Directed leeches to be applied. The next morning he again made his appearance; the leeches had relieved him temporarily, and the disease had apparently gained strength by its resting-spell, for all of the symptoms were aggravated, and the upper lip was beginning to enlarge. Being convinced that suppuration of the nerve was about to take place, we directed warm and emollient applications to be made; these directions were not attended to, and after a good deal of suffering, an abscess opened above the tooth, and the soreness continuing after the disappearance of the more aggravating symptoms, we were requested to operate. This we commenced by drilling through the posterior surface of the tooth into the nerve cavity. The tooth had been filed and filled upon both approximal surfaces, but the filed spaces having closed up, we concluded it would hardly be safe to file them at this time as the jarring of the file would be likely to interfere with the ultimate success of the operation by increasing the inflammation at the root of the tooth.

After removing the remains of the nerve from this tooth, we applied the creosote in the manner described. This application was renewed several times, and as soon as the soreness at the root of the tooth disappeared, we filled the nerve cavity with very finely cut strips of No. 30 gold foil, and then filled the crown cavity with No. 4. Considerable pain was felt upon forcing the first two or three pieces of gold into the extremity of the fang, but as more gold was packed in the uneasiness diminished, and when the operation was finished the tooth felt perfectly free from pain. Shortly after this the abscess healed, but at the end of a year it again appeared, and continued to give more or less trouble for some months.—Finally it vanished, and again appeared in the latter part of 1854. Early in 1855 the patient felt one morning what he supposed to be a bristle from his tooth pricking his upper lip and gum; upon examining it with a glass he discovered something bright. With a little trouble this was pulled out and proved to be one of the small strips of No. 30, used in filling the fang of the tooth.

Here then was a failure—the abscess was not cured. It was not, however, a failure of the medical but of the mechanical treatment. It was an accident that could not have been foreseen, and was doubtlessowing to an unusually large opening at the extremity of the fang. We have understood that since the removal of the piece of gold no trouble has been experienced.

Case III.—Miss L—— consulted us early in January, 1855, relative to a left superior incisor. The tooth had been filled some years before. Arsenic had been used to obtund the sensibility of the bone previous to excavating. After the whole operation was completed, the tooth remained sore for a long time; finally very severe pain, and inflammation was followed by death of nerve, abscess, &c.

When we saw the patient the tooth was very much discolored, and as the lady's teeth were large, regular, and finely-shaped, this change in color detracted most materially from her beauty. There was no opening through the alveolus above, or in the neighborhood of the tooth, nor had there been any for nearly two years. There was, however, a hard and apparently bony tumor as large round as a ten cent piece, and elevated at its centre about one-fourth of an inch beyond the level of the gum. This tumor was at times quite painful, and the tooth often felt quite loose. The greatest annoyance to the patient was the protrusion of the lip and the discoloration of the tooth. The two combined producing a decidedly unpleasant effect upon what would otherwise have been a very pretty and interesting face.

Upon examination the pulp cavity was found to be filled with a dark green semi-liquid, and horribly offensive matter. This was as thoroughly removed as possible, and the tooth repeatedly washed out with tepid water and dried with floss cotton. After which, the usual application of creosote and floss silk was allowed to remain twenty-four hours, and then, after washing out the canal, applications were renewed. This treatment was persisted in for about ten days, when all trace of the odor of decomposition had disappeared, and the tumor at the extremity of the fang had become diminished by about one-half. The fang was now filled, and at the end of twenty-four hours no unpleasant symptoms having appeared, the operation was completed by filling the crown cavity. Removing the decomposed contents of the nerve cavity had sensibly restored the color of the tooth, but after cutting away a thin layer of bone forming the inner wall of the nerve cavity, the change was more perceptible. The color was still farther restored by filling the crown cavity with gold. We did not see our patient again for several weeks, and then had the pleasure of knowing that the tumor had entirely disappeared. There had been no unpleasant symptoms connected with the tooth. The color remained the same as when filled, and it would require a very close examination to detect any difference between this tooth and its fellows.—*Dental Recorder.*

[TO BE CONTINUED.]

